# Computational Analysis of Fluid Forces on an Obstacle in a Channel Driven Cavity: Viscoplastic Material Based Characteristics

**DOI:** 10.3390/ma15020529

**Published:** 2022-01-11

**Authors:** Rashid Mahmood, Afraz Hussain Majeed, Qurrat ul Ain, Jan Awrejcewicz, Imran Siddique, Hasan Shahzad

**Affiliations:** 1 Department of Mathematics, Air University, PAF Complex E-9, Islamabad 44000, Pakistan; rashid.mahmood@mail.au.edu.pk (R.M.); qurratulain_q@yahoo.com (Q.u.A.); 2 Department of Automation, Biomechanics and Mechatronics, Lodz University of Technology, 1/15 Stefanowskiego, St., 90-924 Lodz, Poland; jan.awrejcewicz@p.lodz.pl; 3 Department of Mathematics, University of Management and Technology, Lahore 54770, Pakistan; imransmsrazi@gmail.com; 4 Faculty of Materials and Manufacturing, College of Mechanical Engineering and Applied Electronics Technology, Beijing University of Technology, Beijing 100124, China; hasanshahzad99@hotmail.com

**Keywords:** viscoplastic material, finite element method, fluid forces, yield stress, channel-driven cavity

## Abstract

In the current work, an investigation has been carried out for the Bingham fluid flow in a channel-driven cavity with a square obstacle installed near the inlet. A square cavity is placed in a channel to accomplish the desired results. The flow has been induced using a fully developed parabolic velocity at the inlet and Neumann condition at the outlet, with zero no-slip conditions given to the other boundaries. Three computational grids, *C*_1_, *C*_2_, and *C*_3_, are created by altering the position of an obstacle of square shape in the channel. Fundamental conservation and rheological law for viscoplastic Bingham fluids are enforced in mathematical modeling. Due to the complexity of the representative equations, an effective computing strategy based on the finite element approach is used. At an extra-fine level, a hybrid computational grid is created; a very refined level is used to obtain results with higher accuracy. The solution has been approximated using *P*_2_ − *P*_1_ elements based on the shape functions of the second and first-order polynomial polynomials. The parametric variables are ornamented against graphical trends. In addition, velocity, pressure plots, and line graphs have been provided for a better physical understanding of the situation Furthermore, the hydrodynamic benchmark quantities such as pressure drop, drag, and lift coefficients are assessed in a tabular manner around the external surface of the obstacle. The research predicts the effects of Bingham number (*Bn*) on the drag and lift coefficients on all three grids *C*_1_, *C*_2_, and *C*_3_, showing that the drag has lower values on the obstacle in the *C*_2_ grid compared with *C*_1_ and *C*_3_ for all values of *Bn*. Plug zone dominates in the channel downstream of the obstacle with augmentation in *Bn*, limiting the shear zone in the vicinity of the obstacle.

## 1. Introduction

Even though the bulk of fluids in the biosphere exhibit Newtonian behavior, most synthetic and nonsynthetic industrial fluids, as well as biological fluids such as the blood, and saliva of humans, exhibit non-Newtonian behavior. Cosmetics, paints, soaps, glues, detergents, and various food items are a few examples. Among these, a substantial class of non-Newtonian materials known as viscoplastic materials or Bingham plastic has a yield stress limit that must be accomplished before considerable deformation can occur. This type of non-Newtonian substance is very important. The viscoplastic fluids include slurries and chocolate, pastes and margarine, mayonnaise, suspension, and others. These ingredients have been used in many sectors and culinary processes and are currently an area of increased scientific interest because of their excellent functionality, practical use, and wide-ranging applicability.

The initial idea of yield stress and its technical aspects was communicated by Shwedov [1], who assessed the behavior of flow, composition, and the quality of viscoplastic materials at various output stress levels. Following Shwedov, an extensive range of experiments was conducted by Bingham, who carried out detailed observations of their fluidity and plasticity [2]. Since then, the interest in these materials has been growing steadily, resulting in numerous studies, modeling, and simulations, that examine such materials in nontrivial flows, either conceptually or experimentally. Several models have such as the Bingham [2], the Herschel–Bulkley [3], and the Casson model [4], attempted to show the relationship between stress and strain in these fluids and gain a better understanding of the physical properties of viscoplastic materials. Thus far, Bingham fluid model seems the strongest; Bingham accomplished remarkable feats by proposing many viscoplastic paints and clarifying the plasticity and fluidity of these materials. Subsequently, Bird et al. [5,6] undertook seminal research, providing a list of several additional materials that exhibit these features. Several attempts [7,8,9] have been made to amend Bingham’s modeling of plastic fluids and create mathematical solutions. Bercovier and Engelmann [7] recognized the discontinuity found in the Bingham model and provided the corrective by linearizing the fluid viscosity. Papanastasiou [8] added to the expression of yield stress by introducing an exponential term. This addition to the model aids in describing yielded and unyielded areas. Barnes [9] provided a detailed study of fluid viscoplastic behavior, stating that the behavior of real viscoplastic fluids is more similar to the Papanastasiou’s regularized viscoplastic fluid than the behavior of ideal Bingham fluid. Further research on yield-stress fluids can be found in [10,11,12,13,14,15].

Much research has been dedicated to the theoretical and practical aspects of the channel flow using Bingham fluids. For the system overriding the motion, the weak solutions have been addressed in [16], and in pipes and plane channel Poiseuille flow, nonlinear stability has been investigated in [17]. For the problem of incoming flow in a pipe, spatial decay estimations have been studied in [18], and slip conditions are used to study Couette–Poiseuille flow in a porous channel in [19]. The convective flow of Bingham fluid in a vertical channel has also been studied; for the Couette–Poiseuille flow, the natural convection is studied in [20]. In a porous channel, the influence of exterior and internal heating on the free convective flow has been explored using Pascal’s piecewise-linear law for Poiseuille flow in [21] and in [22] for mixed convection of non-Newtonian fluids, the analytic solutions have been produced.

The flow around obstacles is a fascinating fluid mechanics problem from a computational, experimental, and analytical standpoint. Due to technological developments in recent years, difficulties with the computational and temporal issues associated with simulations around obstacles have been eliminated. Researchers nowadays focus their efforts on gaining a better knowledge of fluid movement around obstacles and interpreting it physically. Schaefer et al. [23] carried out ground-breaking work in this area by studying the flow characteristics of Newtonian fluid flow around an obstacle. Outcomes for Newtonian fluid throughout the cylinder accompanied by different physical necessities can be found in [24,25,26,27]. The literature includes limited work on non-Newtonian fluids flowing around obstacles. C. H. K. Williamson [24] investigated the characteristics of vortex shedding over a bluff body. Hussain et al. [25] considered continuous and discontinuous Galerkin approaches for computing incompressible problems and investigated the control of the mean flux. Kanaris et al. [26] studied the features of the 3D flow field over an obstacle in a channel and analyzed the confinement only in far wake. Rajani et al. [27] have focused on describing the viscous flow over a cylinder in a channel. The main findings are validated against skin friction and Strouhal frequency shedding. Adachi and Yoshioka [28] investigated the theoretical work in this area. Tokpavi et al. [29,30] examined Bingham fluids around the circular cylinder, including inertia effects. They examined the flow characteristics of Bingham fluid experimentally and showed excellent agreement between earlier theoretical conclusions and their experimental data. The Bingham fluid was exploited by Nirmalkar et al. [31] to explain the forced thermal convection effect past a square shape cylinder. The drag and lift coefficients magnitudes were determined for Reynolds number (*Re*) = 45 by Mossaz et al. [32] and Syrakos et al. [33] for different values of Bingham number. Moreover, Syrakos et al. [34] investigated the idea of an effective Reynolds number for Bingham fluid.

Abbasi et al. [35] quantitatively evaluated the effects of fluid forces using a cylinder-based Lattice Boltz–Mann Method (LBM) at low Reynolds numbers. Using $P_{2}-P_{1}$ element pair for finite element approach to a channel-driven cavity, Mahmood et al. [36] examined the non-Newtonian flow. Khan et al. [37] used the COMSOL solver to impose least-square FEM computation of viscous fluid flow through a semicylinder block. To tackle a flow problem, Tomio et al. [38] constructed a numerical approach, second-order, finite difference schemes. Recently, Afraz et al. [39] provided a full investigation of fluid forces and thermal analysis of two-dimensional, incompressible, and laminar complicated fluid flow.

The flow of Bingham material has been investigated by many researchers both computationally and experimentally. However, Bingham flow in the present configuration in the presence of obstacles is new. The discussion below is organized as follows; in Section 2, mathematical modeling is explained. The physical configuration and numerical approach are executed in Section 3. A detailed analysis of results is considered in Section 4, and the conclusion is offered in Section 5.

## 2. Mathematical Modeling

In dimensional form, the model Equations for steady, incompressible flows are defined in [15] as follows:

(1) ∇.u=0

(2) ρ(u.∇u)=−∇p+∇.τ

where all the symbols have their traditional meanings. Bingham [2] developed a basic rheological relationship for viscoplastic materials:

(3) $$\{\begin{array}{l} \;\;\;\;\begin{array}{l} \dot{\gamma }=0,\;\;\;\;\;\;\;\;\;\;\;\;\;\;\;\;\;\;\;\tau \leq \tau_{y}\\ \tau =\left(\frac{\tau_{y}}{\dot{\gamma }}+\mu_{P}\right),\;\;\tau >\tau_{y} \end{array}\; \end{array}$$

where the $\tau ,\;\dot{\gamma },\;\tau_{y}\;\mathrm{and}\;\mu_{P}$ denotes the stress tensor, the rate of the strain tensor, the yield stress, and plastic viscosity, respectively. The strain tensor is defined as

(4) $$\dot{\gamma }\equiv \nabla u+{\left(\nabla u\right)}^{T}$$

Here, u denotes the velocity vector. Defining stress magnitude and strain rate as

(5) $$\tau \equiv {\left[\frac{1}{2}\left(\tau \right):\left(\tau \right)\right]}^{\frac{1}{2}},\;\;\;\;{\dot{\gamma }}^{.}\equiv {\left[\frac{1}{2}\left(\dot{\gamma }\right):\left(\dot{\gamma }\right)\right]}^{\frac{1}{2}}$$

One crucial observation is that the computational domain can be divided into three areas for viscoplastic fluids, the first where $\dot{\gamma }\neq 0$ defines the shear zone, whereas $\dot{\gamma }\equiv 0$ shows plug zone and U≠0. There is a discontinuity inherited by Equation (3) which is addressed by Papanastasiou [8] using exponential function as

(6) $$\tau =\left[\frac{\tau_{y}}{\dot{\gamma }}\left\{1-exp\left(-m\dot{\gamma }\right)\right\}+\mu_{P}\right]\dot{\gamma }$$

Here, the parameter m denotes the stress growth. The viscosity, by owing Equation (4), can be written as

(7) $$\eta =\left[\frac{\tau_{y}}{\dot{\gamma }}\left\{1-exp\left(-m\dot{\gamma }\right)\right\}+\mu_{P}\right]$$

Which for the entire flow domain is valid.

Introducing, $u^{*},\tau^{*},{\dot{\gamma }}^{*}\;\mathrm{and}\;p^{*}$ the nondimensional variables and choosing $L_{ref}$ and $U_{ref}$ as reference length and velocity, respectively, such that

(8) $$\nabla .u^{*}=0$$

(9) $$Re\;u^{*}.\nabla u^{*}=-\nabla p^{*}+\nabla .\tau^{*}$$

In which

(10) $$\tau^{*}=\left[\frac{\tau_{y}}{{\dot{\gamma }}^{*}}\left\{1-exp\left(-M{\dot{\gamma }}^{*}\right)\right\}+1\right]{\dot{\gamma }}^{*}$$

where $Re=\frac{\rho U_{ref}L_{ref}}{\mu_{P}}$ is Reynolds number and Bingham number is $Bn=\frac{\tau_{y}L_{ref}}{\mu_{P}U_{ref}}$. The parameter m is now given by $M=\frac{mU_{ref}}{L_{ref}}$. The nondimensional form of viscosity is

(11) $$\eta^{*}=\left[\frac{Bn}{{\dot{\gamma }}^{*}}\left\{1-exp\left(-M{\dot{\gamma }}^{*}\right)\right\}+1\right]$$

*M* is the nondimensional correspondence of *m.*

The hydrodynamic forces are accessible, the drag coefficient and lift coefficient $C_{D}$ and $C_{L}$ are immediately available for postprocessing by a nondimensional analog

(12) $$C_{D}=\frac{2F_{d}}{\rho U_{mean}^{2}D}$$

(13) $$C_{L}=\frac{2F_{l}}{\rho U_{mean}^{2}D}$$

Here, $U_{mean}$ denotes reference velocity, and *D* is the diameter of the obstacle.

## 3. Physical Configuration and Numerical Scheme

**Figure 1:**
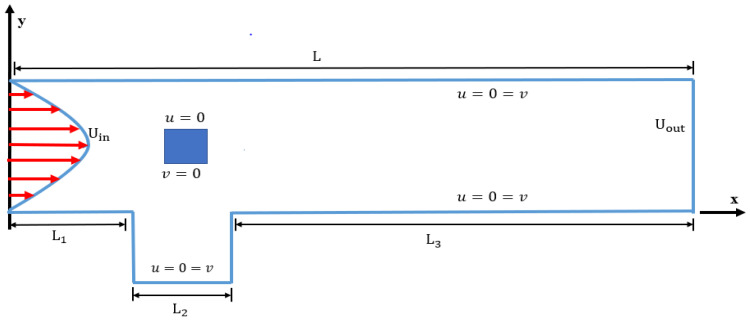
Physical configuration of the problem.

A schematic diagram of the channel-driven cavity is shown in Figure 1. An open square cavity is placed at the bottom of the channel. A parabolic inflow profile is provided at the channel’s inlet, and at the outlet, a Neumann condition is addressed. The other walls of the channel-driven cavity is set zero no-slip condition, i.e., u=v=0. For more accuracy of hydrodynamic forces, an extra-fine hybrid mesh is developed around the obstacles. A square shape cylinder is placed by varying the locations with centers respectively, (0.9, 1.5), (1.5, 1.5), and (2.1, 1.5).

Considering incompressible Navier–Stokes equations given in (1) and (2), together with the rheological law (3) representing Bingham material, a broad range of flow problems can be described. These equations describe the natural processes of life and contribute to understanding the flow of materials in nature. Due to the high nonlinearity of the model, exact solutions to such problems are rare; therefore, we apply FEM computation for the numerical approximation of the governing equations. In this direction, the conforming element pair ${\mathbb{P}}_{2}-{\mathbb{P}}_{1}$ is selected for the velocity and pressure approximations. This element is a stable pair satisfying the inf–sup condition [40,41,42,43]. Newton’s method is applied to solve discrete nonlinear algebraic systems, and the inner linear subproblems are solved using a direct solver. The nonlinear iteration’s convergence condition is specified as follows:

$$\left|\frac{X^{n+1}-X^{n}}{X^{n+1}}\right|<10^{-6}$$

where X denotes the general component of the solution.

**Figure 2:**
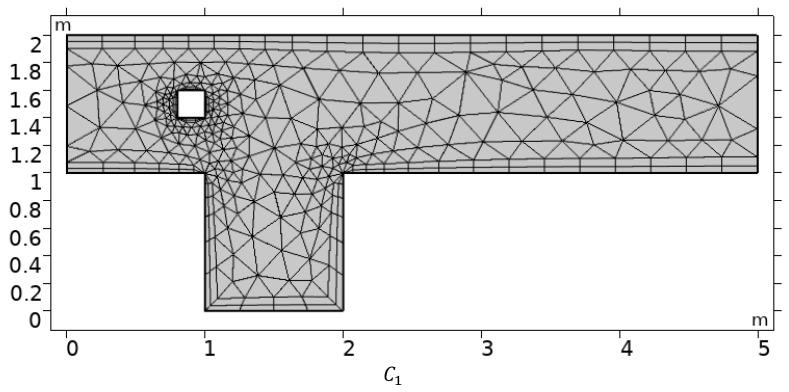

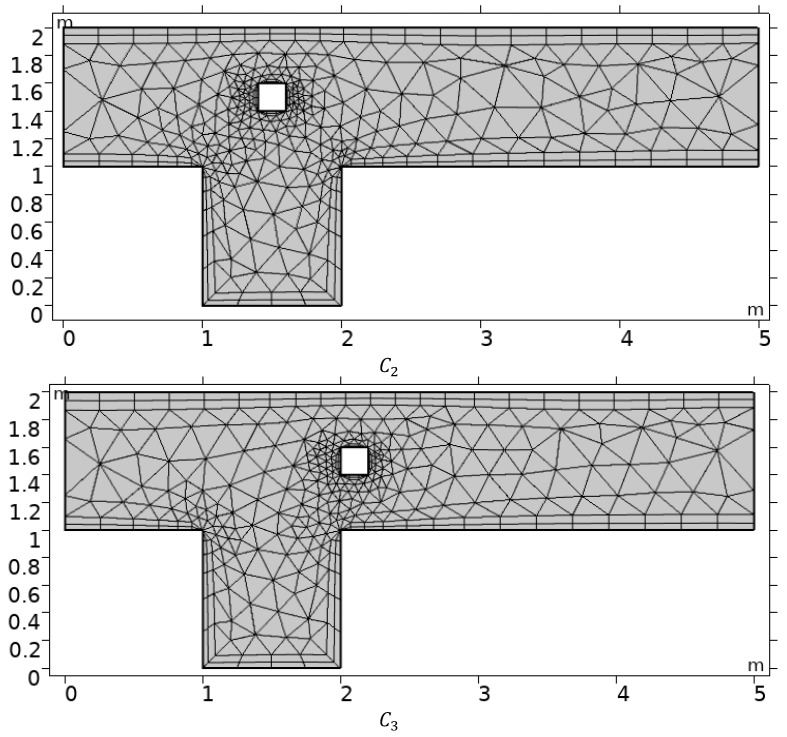
Computational coarse meshes for different positions of obstacle.

The coarse computational grid for three different obstacle settings $C_{1},C_{2},$ and $C_{3}$ are shown in Figure 2. Mesh refinement is an important step in validating any finite element model and enhancing the reader’s trust in the work’s physical outcomes. Since the finite element method (FEM) is based on a transformation of computational domain into the finite number of elements, in Table 1, mesh statistics at different refinement levels for several elements (# EL), and associated degrees of freedom (# DOF) are enumerated.

## 4. Results and Discussions

**Figure 3:**
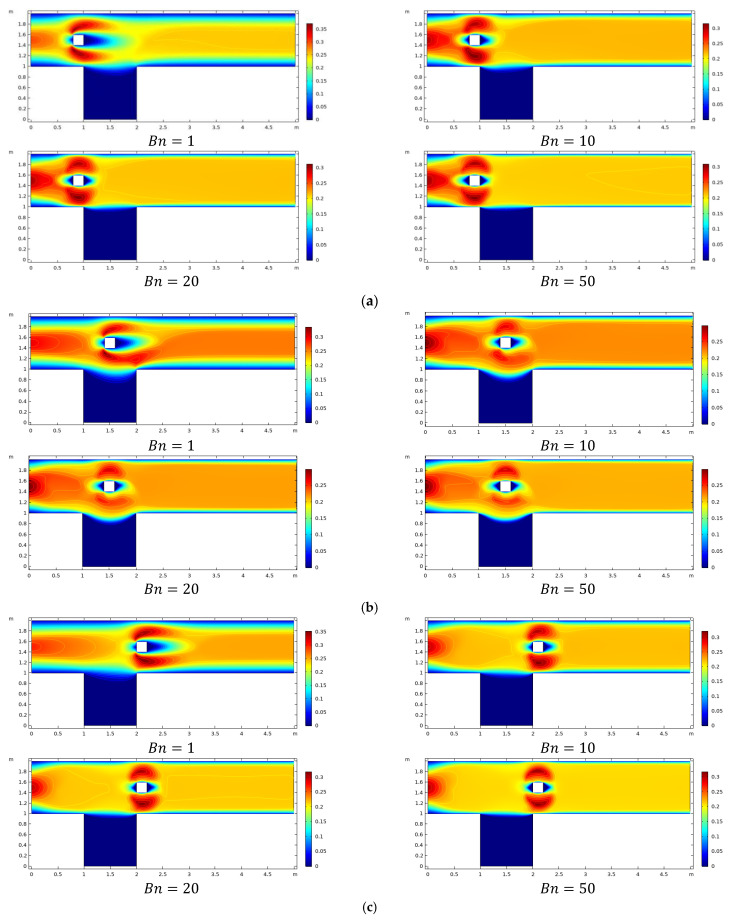
(**a**): Velocity profile with the obstacle center at *C*_1_ for various *Bn*. **(b**): Velocity profile with the center of obstacle at *C*_2_ for various *Bn*. (**c**): Velocity profile with the center of obstacle at *C*_3_ for various *Bn*.

Figure 3a–c illuminates momentum distribution by adjusting Bingham number from Bn = 1 up to 50. The position of the square obstacle is changed, centered at different $C_{1},C_{2},$ and $C_{3}$. The Bingham fluid flow is seen at a fixed Reynolds number Re=20. Fluctuation in velocity near an obstacle and other regions of the channel-driven cavity is noticed because the induced velocity at the inlet is parabolic, while for other boundaries, there is a no-slip condition. For all locations $C_{1},C_{2},$ and $C_{3},$ it is observed that velocity decreases with an increase in Bn, and the plug zone stretches from the channel’s center to the solid walls, while the shear zone is limited to the immediate vicinity of obstacles.

**Figure 4:**
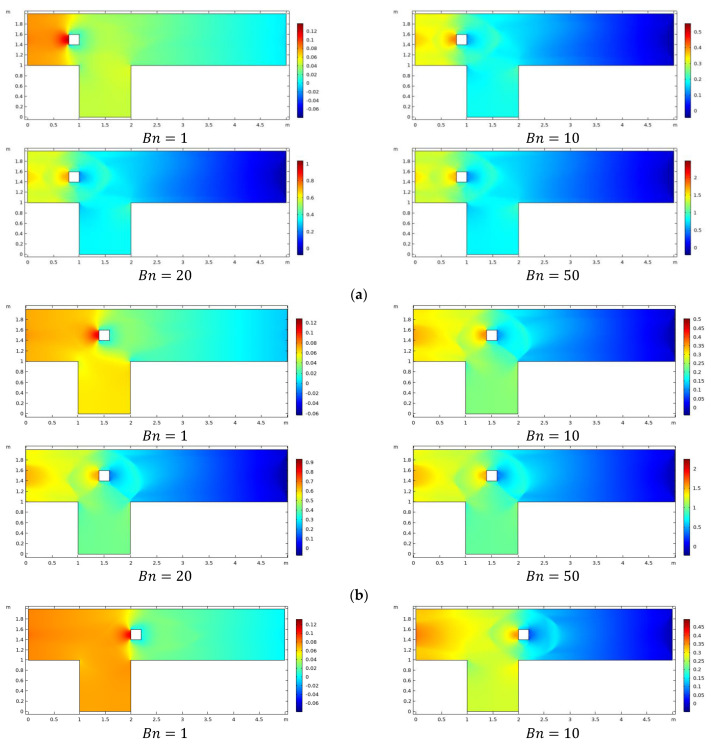

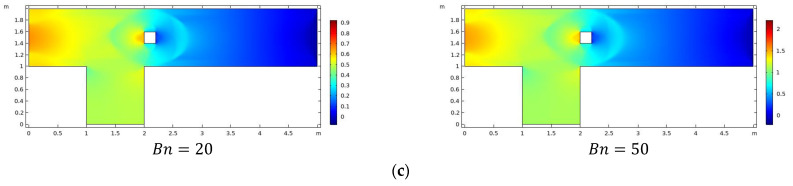
(**a**): Pressure when the center of obstacle at *C*_1_ for various *Bn*. (**b**): Pressure when the center of obstacle at *C*_2_ for various *Bn*. (**c**): Pressure when the center of obstacle at *C*_3_ for various *Bn*.

The pressure changes across the physical domain, particularly in the presence of obstacles located at three different sites by varying Bn, for restricted value Re=20, is plotted in Figure 4a–c. The figure illustrates that pressure behaves nonlinearly near the obstacle before becoming linear downstream, as predicted in channel flow. Because of plasticity effects amplified by enhancement in the value of Bn, the parabolic profile given at the inlet quickly bifurcates at the obstacle and subsequently decreases in the center of the channel. The maximum value of optimal pressure is observed in the presence of an obstacle that interacts with the fluid.

**Figure 5:**
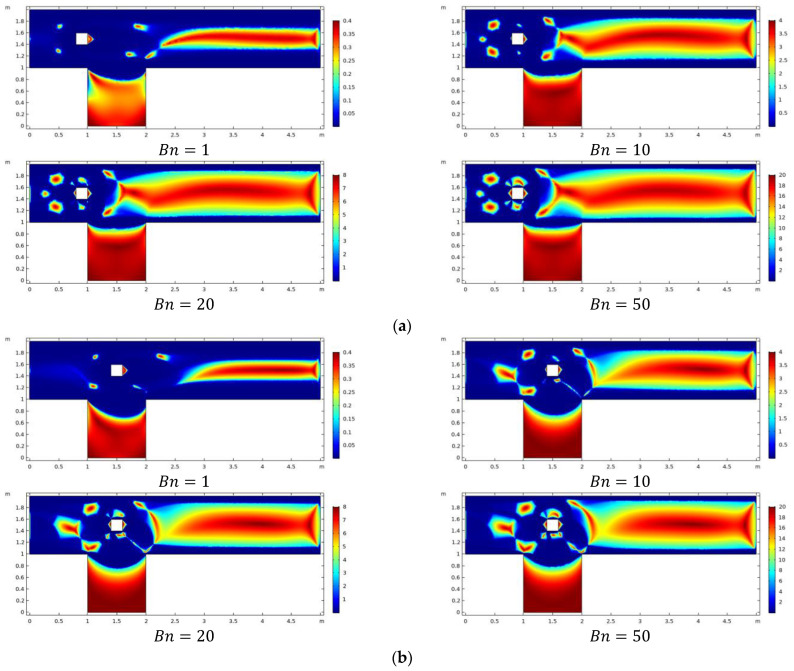

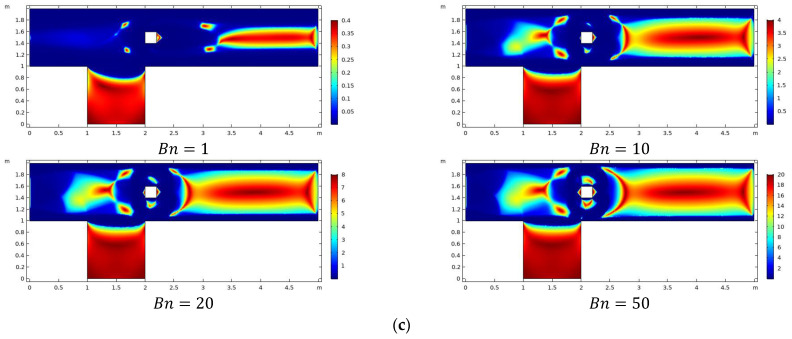
(**a**): Viscosity with the obstacle center at *C*_1_ for various *Bn*. (**b**): Viscosity with the center of obstacle at *C*_2_ for various *Bn*. (**c**): Viscosity with the center of obstacle at *C*_3_ for various *Bn*.

The influence of relevant parameters on viscosity is plotted in Figure 5a–c. By increasing the magnitude of Bn, an increase in viscosity is also observed. Higher viscosity values are seen as Bn increases even for different obstacle positions. In addition, around the obstacle, there are small islands of high viscosity for all obstacle sites, and their size is growing with increasing Bn.

**Figure 6:**
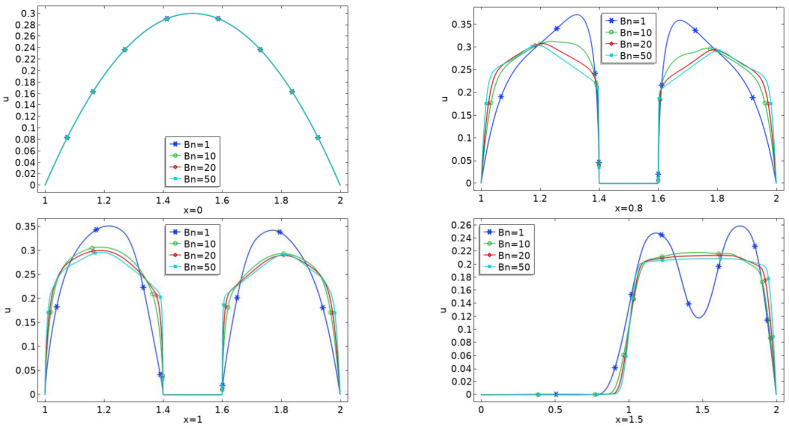

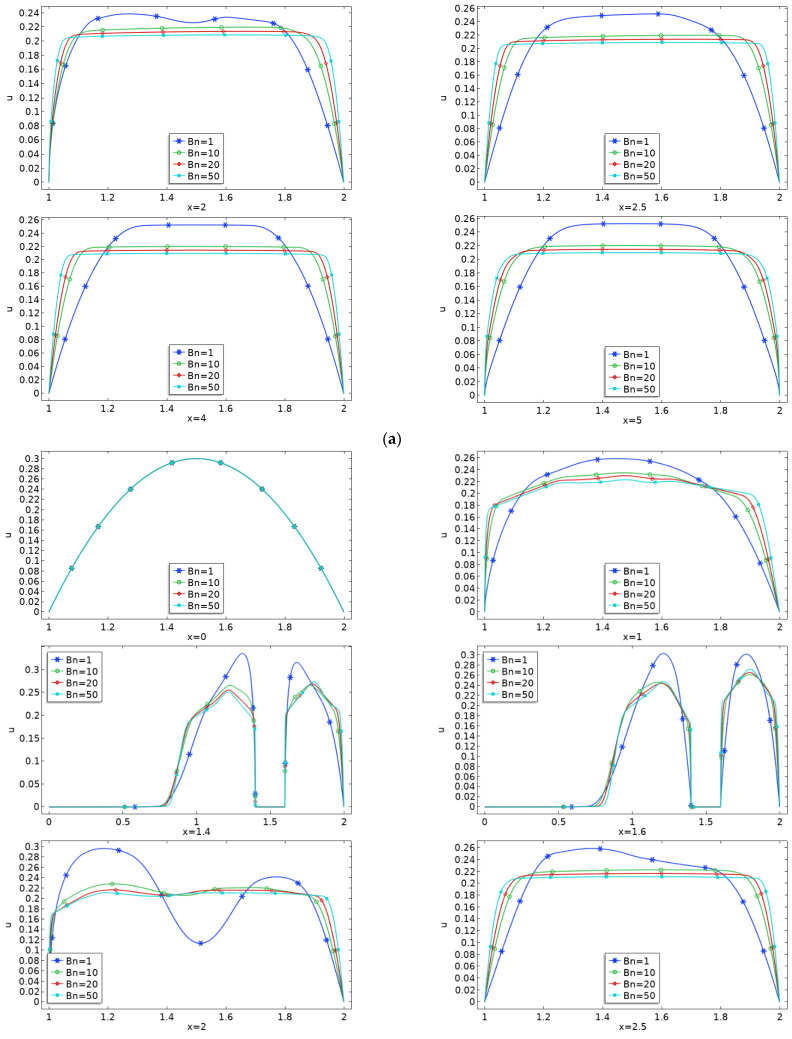

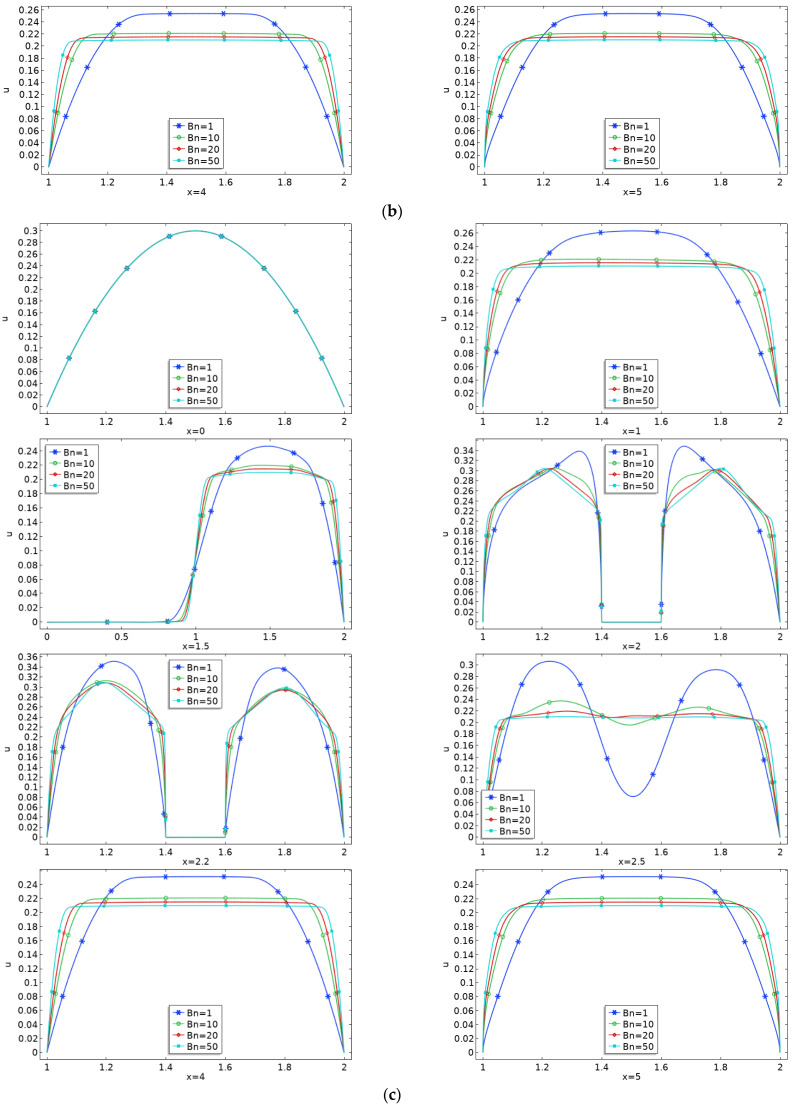
(**a**): Line graph of velocity profile when the center of obstacle at *C*_1_ for various *Bn*. (**b**): Line graph of velocity profile when the center of obstacle at *C*_2_ for various *Bn*. (**c**): Line graph of velocity profile when the center of obstacle at *C*_3_ for various *Bn*.

Figure 6a–c depicts line graphs expressing velocity variations at various points in the physical configuration. Because viscosity does not affect injected velocity, a perfect parabolic peak is achieved at the inlet, as seen in the above illustrations. The fluid flow through the channel, on the other hand, varies its behavior near the obstacle and above the cavity. A gradual rise in nonlinearity can also be detected due to Bn growth.

For increasing values of Bingham number (Bn) and confining Re=20, Table 2 interprets fluctuation in the pressure across obstacles placed at distinct points in the channel, such as the centers at $C_{1},C_{2},$ and $C_{3}$. It is enumerated from the numerical data obtained by increasing Bn pressure drop. The viscosity of Bingham fluids increases as Bn increases, causing it to contact the obstacle with greater force and thereby increasing pressure drop. The numeric data obtained for Bn ranging from (1 to 50) indicates that the pressure gradient approaches 1.144143 at the obstacle’s position $G_{1}$ and approaches 1.129823 and 1.164679 at the obstacle’s positions $C_{2}$ and $C_{3}$, respectively. It is also observed that the obstacle’s position affects the pressure gradient. The manipulated difference is evidenced by tabulated values at Bn=1 for restriction Re=20, for location $C_{1}$ of the obstacle, the pressure drop value is 0.105098, whereas for the same value of Bn and Re at the other two locations, i.e., at $C_{2}=0.089917$ and $C_{3}=0.102492$ variation in the pressure drop is observed. These findings show that the maximum pressure near the inlet pressure falls over the cavity pressure drops with low intensity, followed by the elevation after the fluid crosses from the cavity.

Table 3 depicts changes in benchmark hydrodynamic parameters such as drag coefficient and lift coefficient on the exterior surface of the obstacle located at $C_{1},C_{2}$ and $C_{3}$. It is found that increasing the scale of Bn drag and lift, coefficients fluctuate for a fixed value of Reynold number Re=20. The negative lift coefficient ($C_{L}$) value illustrates that the lift forces are dominant in the upward direction. The cause for the negative value of lift coefficient is because the obstacle is positioned as if fluid enters the cavity and pushes the obstacle upward, resulting in a numerical pattern like this. From calculated values of drag coefficient at $C_{1},C_{2},$ and $C_{3}$, it is realized that the maximum value of drag is 6.5114676 at $C_{1.}$ By fixing Bn=1 and Re=20 as it comes closer to $C_{2}$, drag value starts to decrease, having a magnitude of 5.71877 and again increases at $C_{3}$ as the magnitude is 6.6158896. The lift coefficient’s contrasting behavior is adjusted so that fluctuation occurs at $C_{1},C_{2},$ and $C_{3}$ as the magnitude of lift coefficient at $C_{1}=-0.24909,$ at the location $C_{2}$ decreased value is noticed of magnitude −0.37351 and again increased to 0.28946 at $C_{3}$. Because upward lift forces dominate, negative values are measured when the obstacle is positioned near the inlet and above the cavity position, while positive values are measured at $C_{3}$ because lift forces are active from downward.

## 5. Conclusions

The current study investigates the flow characteristics of a generalized Bingham material flow in a channel-driven cavity, with an obstacle of square shape positioned at various locations inside the channel. The physical problem and relevant rheological laws are transformed into a mathematical form using the Navier–Stokes equation in two-dimension and boundary constraints. The finite element method, a well-known computational tool, is required to report both the solution and the physical occurrences. Graphical trends are used to present the results. The behavior of the momentum distribution of a Bingham fluid at entrance, obstacle, and close to exit regions is represented by bar lines. The pressure variation, as well as the drag coefficient and lift coefficient on the external surface of the obstacle, are tabulated. The most important findings are listed below.

- As the *Bn* increases, pressure drops become more vigorous for all obstacle positions.
- The pressure drop is influenced by the placement of the obstacle, which increases at *C*_1_, lowers at *C*_2_, and finally boosts at *C*_3_.
- Lift coefficient changes sign-on *C*_2_ when *Bn* exceeds the value *Bn* = 10 while on other grids, sign change does not occur.
- Negative lift coefficients are obtained when upward forces dominate, while positive values are obtained when downward forces dominate.
- Pressure has stagnant values at the front of the obstacle where fluid is interacting with it.
- Plug zone enhances in the channel downstream of the obstacle with augmentation in *Bn* limiting the shear zone in the vicinity of the obstacle.

## Figures and Tables

**Table 1:** Number of Degrees of Freedom at Different Refinement Levels.

Refinement Level	# EL	# DOF
$L_{1}$	769	4032
$L_{2}$	1167	6130
$L_{3}$	1786	9245
$L_{4}$	2962	15,171
$L_{5}$	3590	21,648
$L_{6}$	6707	33,213
$L_{7}$	16,139	78,925
$L_{8}$	39,724	191,180
$L_{9}$	52,844	250,024

**Table 2:** Pressure drop for different positions of obstacle.

*Bn*	*C*_1_	*C*_2_	*C*_3_
	*δp* = *p*_2_ − *p*_1_	*δp* = *p*_2_ − *p*_1_	*δp* = *p*_2_ − *p*_1_
1	0.105098	0.089917	0.102492
5	0.178537	0.161953	0.182264
10	0.277829	0.260403	0.285607
15	0.379743	0.362457	0.391536
20	0.485614	0.471741	0.498451
25	0.590631	0.580957	0.608244
30	0.698603	0.690398	0.718537
35	0.809055	0.799874	0.828990
40	0.920618	0.909552	0.940283
45	1.032414	1.019508	1.052340
50	1.144143	1.129823	1.164679

**Table 3:** Drag and Lift Coefficients for Various *Bn* with Different Centre of Obstacle.

*Bn*	*C*_1_		*C*_2_		*C*_3_	
	*C_D_*	*C_L_*	*C_D_*	*C_L_*	*C_D_*	*C_L_*
1	6.511467	−0.24909	5.718770	−0.373510	6.615889	0.28946
5	12.31288	−0.61309	11.31525	−0.630990	12.61671	0.35282
10	19.78717	−0.90407	18.61922	−0.219770	20.29534	0.72005
15	27.24873	−1.16007	26.00279	0.395369	27.93196	1.21576
20	34.7233	−1.41867	33.41197	0.909717	35.54671	1.75848
25	42.18845	−1.71279	40.81667	1.348083	43.13843	2.30321
30	49.65995	−2.05944	48.21088	1.743551	50.71272	2.83111
35	57.127	−2.43912	55.59006	2.106661	58.27419	3.33006
40	64.58501	−2.82462	62.96177	2.438490	65.82729	3.80311
45	72.04071	−3.20601	70.31964	2.741542	73.36969	4.25363
50	79.48114	−3.58121	77.67343	3.022520	80.90281	4.68517

## Data Availability

Data is available on request from the corresponding author.

## Nomenclature

$\tau_{y}$	Yield Stress
$\mu_{p}$	Plastic Viscosity
τ	Stress Tensor
$\dot{\gamma }$	Rate of Strain Tensor
u	Dimensional Velocity Vector
$u^{*}$	Dimensionless Velocity Vector
$U_{in}$	Velocity at inlet
$U_{mean}$	Average Velocity
$\dot{\gamma }$	Shear Rate
p	Dimensional pressure
$p^{*}$	Dimensionless pressure
m	Dimensional Stress growth parameter
*M*	Dimensionless Stress growth parameter
Re	Reynold number
Bn	Bingham number
**#** EL	Number of Elements
# DOF	Number of degrees of freedom
$C_{D}$	Drag Coefficient
$C_{L}$	Lift Coefficient
